# A fatal case of bupropion (Zyban) hepatotoxicity with autoimmune features: Case report

**DOI:** 10.1186/1752-1947-1-88

**Published:** 2007-09-18

**Authors:** Fawwaz Humayun, Thomas M Shehab, Joseph A Tworek, Robert J Fontana

**Affiliations:** 1 Department of Internal Medicine, St. Joseph Mercy Health System, Ypsilanti, Michigan, 48197, USA; 2 Section of Gastroenterology, St. Joseph Mercy Health System, Ypsilanti, Michigan, 48197, USA; 3 Department of Pathology, St. Joseph Mercy Health System, Ypsilanti, Michigan, 48197, USA; 4 Department of Internal Medicine, Division of Gastroenterology, University of Michigan Medical Center, Ann Arbor, Michigan 48109, USA; 5 Huron Gastro/Center for Digestive Care, Ypsilanti, Michigan, 48197, USA

## Abstract

**Background:**

Bupropion is approved for the treatment of mood disorders and as an adjuvant medication for smoking cessation. Bupropion is generally well tolerated and considered safe. Two randomized controlled trials of bupropion therapy for smoking cessation did not report any hepatic adverse events. However, there are three reports of severe but non-fatal bupropion hepatotoxicity published in the literature.

**Case Presentation:**

We present the case of a 55-year old man who presented with jaundice and severe hepatic injury approximately 6 months after starting bupropion for smoking cessation. Laboratory evaluation demonstrated a mixed picture of hepatocellular injury and cholestasis. Liver biopsy demonstrated findings consistent with severe hepatotoxic injury due to drug induced liver injury. Laboratory testing was also notable for positive autoimmune markers. The patient initially had clinical improvement with steroid therapy but eventually died of infectious complications.

**Conclusion:**

This report represents the first fatal report of bupropion related hepatotoxicity and the second case of bupropion related liver injury demonstrating autoimmune features. The common use of this medication for multiple indications makes it important for physicians to consider this medication as an etiologic agent in patients with otherwise unexplained hepatocellular jaundice.

## Background

Bupropion (Wellbutrin^® ^or Zyban^®^, GlaxoSmithKline, Greenville, NC) is approved for the treatment of mood disorders and as an adjuvant medication for smoking cessation. Bupropion is a weak inhibitor of the neuronal uptake of norepinephrine, serotonin, and dopamine but is chemically unrelated to other known antidepressant drugs including tricyclic and tetracyclic agents as well as selective serotonin re-uptake inhibitors. The mechanism by which bupropion enhances the ability of patients to abstain from smoking is unknown but may involve central noradrenergic and/or dopaminergic pathways.

Bupropion is generally well tolerated and considered safe. Each year, nearly 25% of individuals prescribed smoking cessation aids are started on bupropion SR and there are an estimated 8.7 million prescriptions of bupropion dispensed each year in the United States. Two randomized controlled trials of bupropion therapy for smoking cessation of seven and nine weeks duration did not report any hepatic adverse events [1]. However, there are three reports of severe but non-fatal bupropion hepatotoxicity published in the literature [2-4]. The aim of this paper is to report the first fatal case of bupropion hepatotoxicity which presented in an unusual manner, with autoimmune features, in a middle aged male who was trying to stop smoking.

## Case presentation

A 55-year-old Caucasian male presented in February 2005 with new onset hematuria, easy bruising, and jaundice. He also reported fevers, nausea with vomiting and fatigue in the week prior to presentation without any associated abdominal pain or pruritus. At presentation, he was afebrile and there was no skin rash, hepatosplenomegaly, asterixis, or stigmata of chronic liver disease but he was deeply jaundiced with scleral icterus and multiple ecchymoses. Initial laboratory tests included a white blood cell count of 13.7 (4.0–10.0 K/UL) with a left shift, hemoglobin 15.5 (13.5–17.5 GM/DL), platelets 200 (140–450 K/UL), AST 1466 (20–57 IU/L), ALT 1459 (21–72 IU/L), total bilirubin 5.3 (0.0–1.5 mg/dl), direct bilirubin 4.9 (0.0–0.8 mg/dl), alkaline phosphatase 219 (30–136 IU/L), INR 13, and prothrombin time 145.8 (10.0–13.5 sec). Serum liver biochemistries were normal 4 months prior (AST 32 IU/L, ALT 40 IU/L, Total Bilirubin 0.5 mg/dl). An abdominopelvic CT scan without contrast was unremarkable.

The patient had a history of mild depression, hypertension, and hyperlipidemia. He denied using intravenous drugs or recent travel or sick contacts. He had discontinued alcohol in 2002 but smoked a half-pack of cigarettes for the past 8 years. He was receiving warfarin for a prosthetic mitral valve since 2002 and had a previously stable and therapeutic INR. His other medications for the past 3 years included metoprolol XL, atorvastatin, and aspirin. Paroxetine had been started shortly after surgery and discontinued in May 2004 but restarted in October 2004 for recurrent depressive symptoms. Bupropion 150 mg bid was started for smoking cessation in July 2004 and was continued up until hospitalization (6 months of treatment). The patient reported never having received bupropion or other anti-depressants beyond the paroxetine previously. He also denied ingesting over the counter products such as acetaminophen or herbals. He had allergies to penicillin and sulfa drugs that caused hives.

**Figure 1:**
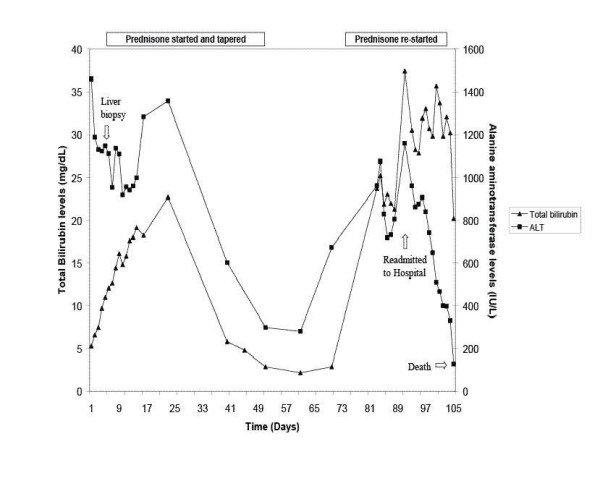
Serial serum alanine aminotransferase and total bilirubin levels. The patient's serum ALT and total bilirubin initially improved with a short course of oral corticosteroids. However, 3 weeks after discontinuing the prednisone, his serum ALT markedly increased and he was rehospitalized. Despite high doses of corticosteroids, he developed progressive mental status changes and died 105 days after initial presentation with sepsis and liver failure.

**Figure 2:**
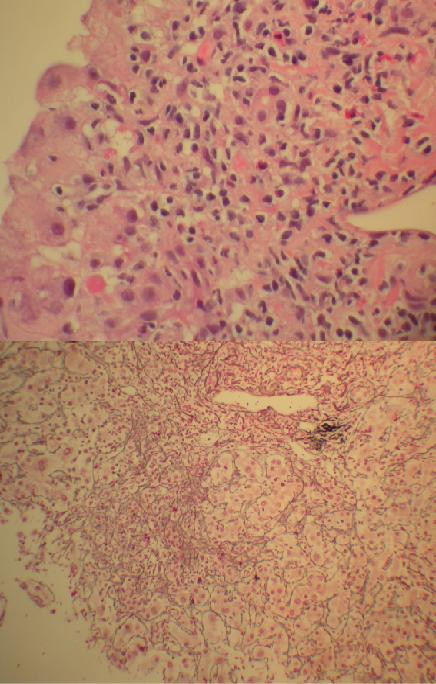
Liver biopsy. (Top) H&E stain showing severe necrosis in the peri-portal region with a mixed population of lymphocytes, eosinophils, and small clusters of plasma cells. (Bottom) Reticulin stain showing crowded reticulin meshwork and drop out of hepatocytes consistent with hepatic collapse. (Magnification of top and bottom, ×400 and ×200).

After receiving several units of fresh frozen plasma, he was temporarily placed on intravenous heparin. Diagnostic studies included a serum iron of 193 ug/dl, transferrin saturation of 55%, and ferritin of 974 mg/dl but subsequent hemochromatosis genotyping was negative. Serum ceruloplasmin was normal at 28 mg/dl. Serological studies for acute hepatitis A IgM, hepatitis B surface antigen and anti-HB core antibody, and hepatitis C RNA by PCR as well as CMV and EBV serologies were negative. However, an anti-nuclear antibody (ANA titer = 1:160; speckled pattern) and anti-smooth muscle antibody (ASMA titer = 1:40) were positive. A surface echocardiogram revealed an ejection fraction of 75–80%. Despite withdrawal of all outpatient medications, his serum aminotransferases and bilirubin continued to rise (Figure 1). A transjugular liver biopsy revealed severe interface hepatitis with intense peri-portal inflammatory infiltrate consisting of a mixture of lymphocytes, eosinophils, and a few scattered plasma cells (Figure 2). A reticulin stain showed hepatic collapse with crowding of the reticulin meshwork and loss of hepatocytes. A trichrome stain did not reveal established fibrosis. A pathological diagnosis of a severe hepatotoxic injury due to a drug with autoimmune-like features was made.

Because of the persistent severe biochemical injury, the patient was started on prednisone 60 mg/day. Over the next 13 days, the serum ALT levels trended down (Figure 1). His total bilirubin peaked at 22.7 mg/dl and his ALT reached a second peak at 1357 IU/L before trending down over the next four weeks. The patient's INR remained difficult to manage even with lower doses of coumadin, ranging between 1.6 and 3.7. However, the INR values became more stable at approximately 20 days after institution of prednisone therapy. Upon referral to the University of Michigan, a repeat ANA was higher at 1:1280 and serum IgG, IgM, and IgA levels were 1510 mg/dl, 125 mg/dl, and 367 mg/dl, respectively. At this point, the patient felt much improved and his prednisone was tapered off over 6 weeks. The patient was discharged on prednisone, metoprolol and coumadin. Three weeks later his transaminases began to rise but his bilirubin remained unchanged. Repeat testing two weeks later showed marked elevation of his total bilirubin to 23.7 mg/dl and ALT to 961 IU/L and he was readmitted to the hospital for a possible repeat liver biopsy. A decision was made to forego the liver biopsy and restart the patient on prednisone 60 mg per day and he was discharged home. However, two days later his total bilirubin increased to 37.4 mg/dl and his ALT was 1158 IU/L. He was then admitted to the hospital for liver transplantation evaluation with new onset mental status changes. The patient was started on broad-spectrum antibiotics. The patient's condition quickly deteriorated with the onset of encephalopathy and coagulopathy. On hospital day 13, he developed respiratory failure and was transferred to the ICU but he died of multiorgan failure the next day. An autopsy revealed coronary artery disease but otherwise intact myocardium. His liver was shrunken and weighed 1320 grams and there was evidence of extensive necrosis, predominantly central zone, with cholestasis. He also had bilateral aspergillus pneumonia, which had previously not been recognized. There was no evidence of other solid organ infection. His death was attributed to sepsis resulting from acute liver failure.

## Conclusion

Bupropion is an effective medication to assist in smoking cessation [1]. Although preclinical studies demonstrated mild, reversible hepatotoxicity in laboratory animals receiving large doses of bupropion for prolonged periods of time [5], initial clinical trials demonstrated an incidence of < 1% of abnormal liver biochemistries. In addition, despite its availability for over 15 years in clinical practice, only 3 cases of bupropion hepatotoxicity have been published in the medical literature [2-4]. In each of these reports, the afflicted subjects had an acute hepatocellular injury pattern developing within 6 months of drug initiation (Table 1). The current patient began bupropion SR for smoking cessation with previously normal liver biochemistries. All of his other medications were longstanding except paroxetine which was restarted in October 2004. Although paroxetine can lead to acute hepatocellular liver injury, the patient had previously received this medication for over 2 years without adverse events. While previous use of the paroxetine could be theorized to have sensitized the patient to retreatment, we feel that the patient's overall clinical course and previously reported case of bupropion-induced liver injury presenting with autoimmune features support our conclusion that bupropion was the most likely inciting agent. Similarly, although warfarin can rarely cause cholestatic liver injury, the continuous use of warfarin for over 3 years makes it unlikely in this instance as well as the hepatocellular liver injury pattern and lack of hypersensitivity features.

**Table 1:** Published reports of bupropion hepatotoxicity

Author (yr)	Dose	Duration of use (Days)	Peak ALT (IU/L)	Peak bilirubin (mg/dl)	Outcome
Oslin ('93)	300 mg QD × 21 days, then 400 mg QD	54	5.4 × ULN	Not reported	Resolution
Hu ('00)	200 mg QD	42	6660	3.8	Resolution
D. Alvaro ('01)	150 mg BID	20	49 × ULN	38	Resolution
Humayun ('07)	150 mg BID	180	20 × ULN	37	Death

ULN = Upper limit of normal

Six months after starting bupropion he presented with markedly elevated serum aminotransferase and bilirubin levels (Figure 1). His time course is consistent with an idiosyncratic drug reaction, which typically occurs within 1 year of starting a new medication [6]. The biochemical profile was a severe acute hepatocellular injury with autoimmune features. Other prescription medications such as minocycline, pemoline, and nitrofurantoin have also been associated with an acute hepatitis with autoimmune features [7]. One prior case of bupropion induced liver injury presented similarly with marked elevation of serum aminotransferase levels and a positive ANA [4]. Our patient was enrolled in the Drug Induced Liver Injury Network (DILIN) prospective protocol wherein all other known competing causes of liver injury were excluded [6]. Using the widely cited Roussel Uclaf Causality Assessment method (i.e. RUCAM), this case scored 8 which is categorized as a "highly probable" case of DILI. This case was also scored as "probable" on a drug-induced hepatitis validation scale [8]. On the other hand, this case was also scored as "probable" autoimmune hepatitis on the International autoimmune hepatitis consensus scale due to the presence of autoantibodies, liver biopsy findings, and initial response to prednisone [9]. Overall, we feel that this patient presumably developed fulminant hepatitis with autoimmune features due to bupropion in light of the temporal course, exclusion of competing etiologies, and compatible liver histology.

"Hy's rule", named after Hyman Zimmerman, has been used to aid in determining the prognosis in cases of DILI. With "Hy's rule" patients with acute hepatocellular DILI and a total bilirubin greater than 2 times the upper limit of normal are anticipated to have a ~10% mortality. This finding was recently validated in a large retrospective series from Sweden [10]. In subjects with severe drug-induced hepatitis that go on to develop encephalopathy and coagulopathy, the likelihood of recovery is even poorer with only a 25% rate of spontaneous survival [11]. Unfortunately, this patient developed a progressive and fatal course despite the cessation of the suspect medication and use of corticosteroids highlighting the utility of "Hy's rule" in identifying DILI patients with a potentially poor prognosis.

The primary management of DILI includes the identification and immediate withdrawal of the inciting agent. There is anecdotal evidence that ursodeoxycholic acid may hasten the resolution of liver biochemical abnormalities but randomized, prospective studies are lacking [12,13]. There are also unproven recommendations of using corticosteroids in immune mediated DILI [4,6]. However, previous controlled trials demonstrated no survival benefit with corticosteroid use in fulminant hepatitis. There is also evidence that steroids may increase the risk of bacterial and fungal infections. In the present case, the patient initially showed significant improvement after the bupropion SR was stopped and corticosteroid treatment was started. The steroids most likely acted by attenuating the ongoing drug induced autoimmune liver damage. Unfortunately, he experienced a severe biochemical relapse after the corticosteroids were tapered which did not respond to retreatment. After progressing to fulminant liver failure, he died with disseminated aspergillosis which was likely related, in part, to the immunosuppressive effect of the corticosteroids. However, fungal infections may develop in 20 to 30% of patients with acute liver failure even in the absence of corticosteroids due to impaired host immune surveillance.

In summary, bupropion is a safe and effective treatment for millions of patients with depression and others seeking to stop smoking. However, as with many other drugs used in clinical practice, there are rare instances of idiosyncratic hepatotoxicity associated with bupropion use. The previously published cases of bupropion hepatotoxicity have all occurred within the first 6 months of medication use and presented with a hepatocellular injury pattern (Table 1). Based upon the clinical presentation, histological findings and time course, it is our opinion that this case is the first reported instance of fatal bupropion hepatotoxicity. Although bupropion hepatotoxicity is rare and unpredictable, practicing physicians should be aware of this adverse effect when evaluating patients with unexplained jaundice or liver biochemistry abnormalities. In addition, bupropion should be considered as a precipitating cause of an acute autoimmune-like hepatitis as has been reported with other prescription medications.

## Abbreviations

AST; Aspartate Aminotransferase, ALT; Alanine Aminotransferase, INR; International Normalized Ratio, PCR; Polymerase Chain Reaction, CMV; Cytomegalovirus, EBV; Epstein-Barr virus, ANA; Antinuclear Antibodies.

## Competing interests

The author(s) declare that they have no competing interests.

## Authors' contributions

FH established diagnosis and medical treatment. TS participated in patient care and the drafting of the manuscript. JT read the original pathology. RJF participated in patient care and drafting of the manuscript. All authors read and approved the final manuscript.
